# Editorial: Organizations between continuity and disruption – The organization and management of perpetual change in times of digitalization

**DOI:** 10.3389/fsoc.2025.1758521

**Published:** 2026-01-05

**Authors:** Thomas Wendt, Inga Truschkat

**Affiliations:** 1 University of Trier, Trier, Germany; 2 Freie Universitat Berlin, Berlin, Germany

**Keywords:** artificial intelligence, digital transformation, digitalization, organizational education, theory of organization

The question of how societal change unfolds has accompanied organizational research since its earliest beginnings. Viewed across a historical trajectory, this development reveals a persistent dynamism: new terms, models, and conceptual tools are continuously introduced to enhance theoretical resolution, deepen understanding of organizational structures, and make processes of change more analytically and practically manageable. This applies equally to management and governance concepts and to evolving understandings of leadership—whether in for-profit, non-profit, or public-sector contexts. From this perspective, the history of organization, management, and leadership itself appears as a process of ongoing transformation: change is the norm.

Current processes of digitalization raise the question of whether—and in what ways—this normality of organizational change is continuing or being fundamentally reshaped. Digitalization and the diffusion of artificial intelligence create technical possibilities that profoundly transform organizations (Kette and Tacke, 2021). Algorithms increasingly define spaces of action and decision-making (Wendt, 2021), placing renewed emphasis on formal structures (du Gay, 2020), which both gain importance and become subject to new forms of relativization. Digital systems operate with considerable autonomy and shape social processes in their own right (Manhart and Wendt, 2021). At the same time, digital infrastructures generate new modes of interaction within organizations (Bormann and Truschkat, 2022), and it is becoming clear that human perceptual and creative capacities can be substituted by technological systems. It is therefore unsurprising that “fluid,” “temporary,” “virtual,” and “unconventional” organizational forms (Besio and Tacke, 2023) increasingly coexist with classical forms, prompting renewed questions about governance logics and the diffusion of organizational concepts (Bromley and Meyer, 2021). Against this backdrop, this Research Topic assembles contributions that illuminate these dynamics and advance organizational research on digitalization.

The opening contribution provides a conceptual reconstruction of the foundational shifts that make digitalization possible in the first place. Manhart demonstrates how societal practices of measuring, calculating, and datafication have historically differentiated themselves from communication, establishing a new semiotic order of digital intelligence—an order that simultaneously produces specific organizational and educational conditions for subjective learning and agency. This perspective on the deep structures of digital culture complements the empirical and theoretical insights presented in the subsequent contributions.

Building on this, Besio et al. examine the opposite pole of organizational dynamics. Using the case of a German public administration, they show that not only rigidity but also ongoing and uncoordinated change can impede digital transformation. Their concept of organizational restlessness highlights that sustainable digitalization requires a nuanced balance between stability and change, challenging long-standing assumptions about bureaucratic organizational logic.

Maack extends these considerations by theoretically reframing the boundary between organization and digitality. Drawing on neo-materialist approaches, she illustrates how relational entities emerge within digitalized settings, transforming the meaning of organizational practices themselves. In this perspective, digitalization appears not merely as an external influence but as an ontological dynamic through which organizations and technologies mutually constitute one another.

The historical–semantic dimension of organizational change is taken up by Freis, who reconstructs shifting emotional expectations within organizational contexts. She demonstrates how modern organizations increasingly require their members to regulate emotions and engage in empathic communication—processes that are intensified by digital transformation. From this angle, emotions do not stand in opposition to rationalization but emerge as historically evolved organizational principles that are reconfigured through digitalization.

A different angle on the logic of collective action in the digital age is offered by Schröder, who applies the concept of transpoiesis to analyze social movements. Using the example of the World Social Forum, he shows how movements negotiate the tension between decentralization and strategic coherence while adapting digital communication formats. His analysis demonstrates that digitalization not only transforms formal organizations but also networked collectives whose learning and identity formation require new theoretical perspectives.

Questions of social inequality are foregrounded by Vollmar, who proposes an organizational-theoretical shift in perspective. She argues that digital inequalities always emerge in organization-specific ways because digital practices and infrastructures are embedded in concrete organizational settings. This reframing redirects analytical attention from technology-centered to organization-centered mechanisms of inequality production and introduces a methodological framework that reshapes the study of digitalized work.

Focusing more closely on leadership theory, Freis and Schröer examine how concepts of digital leadership further relativize classical leadership models under conditions of increasing complexity. Drawing on discourse analysis and initial empirical insights from meta-organizations, they show that digital leadership should be understood less as a rupture than as an extension of unheroic leadership concepts that place greater emphasis on informal structures and processes of subjectivization.

The Research Topic concludes with a contribution by Peters and Truschkat, who investigate social start-ups as novel actors within the digitalization discourse of social services. Due to their hybrid structures and inherently digital organizational modes, these organizations significantly shape discourses, practices, and innovation pathways in the field. Their analysis demonstrates how such organizational forms introduce alternative interpretations and models of socio-digital practice that challenge established providers.

Taken together, the contributions offer a multifaceted perspective on digitalization as an organizational, cultural, social, and semantic transformation process. The Research Topic thus positions itself as an invitation to understand digitalization research more explicitly and more profoundly as organizational research.
